# Resuscitation arterial waveform quantification and outcomes in pediatric bidirectional Glenn and Fontan patients

**DOI:** 10.1038/s41390-024-03564-y

**Published:** 2024-09-16

**Authors:** Andrew R. Yates, David A. Hehir, Ron W. Reeder, John T. Berger, Richard Fernandez, Aisha H. Frazier, Kathryn Graham, Patrick S. McQuillen, Ryan W. Morgan, Vinay M. Nadkarni, Maryam Y. Naim, Chella A. Palmer, Heather A. Wolfe, Robert A. Berg, Robert M. Sutton, ICU-RESUS, Andrew R. Yates, David A. Hehir, Ron W. Reeder, John T. Berger, Richard Fernandez, Aisha H. Frazier, Kathryn Graham, Patrick S. McQuillen, Ryan W. Morgan, Vinay M. Nadkarni, Maryam Y. Naim, Chella A. Palmer, Heather A. Wolfe, Robert A. Berg, Robert M. Sutton, Tageldin Ahmed, Michael J. Bell, Robert Bishop, Matthew Bochkoris, Candice Burns, Joseph A. Carcillo, Todd C. Carpenter, J. Michael Dean, J. Wesley Diddle, Myke Federman, Ericka L. Fink, Deborah Franzon, Stuart H. Friess, Mark Hall, Christopher M. Horvat, Leanna L. Huard, Tensing Maa, Arushi Manga, Kathleen L. Meert, Peter M. Mourani, Daniel Notterman, Murray M. Pollack, Anil Sapru, Carleen Schneiter, Matthew P. Sharron, Neeraj Srivastava, Sarah Tabbutt, Bradley Tilford, Shirley Viteri, David Wessel, Athena F. Zuppa, the Eunice Kennedy Shriver National Institute of Child Health and Human Development Collaborative Pediatric Critical Care Research Network Investigator Groups, Andrew R. Yates, Ron W. Reeder, John T. Berger, Kathryn Graham, Patrick S. McQuillen, Robert A. Berg, Robert M. Sutton, Michael J. Bell, Joseph A. Carcillo, Todd C. Carpenter, J. Michael Dean, Ericka L. Fink, Mark Hall, Kathleen L. Meert, Peter M. Mourani, Daniel Notterman, Murray M. Pollack, Anil Sapru, David Wessel, Athena F. Zuppa

**Affiliations:** 1 Department of Pediatrics, Nationwide Children’s Hospital, The Ohio State University, Columbus, OH USA; 2 Department of Anesthesiology and Critical Care Medicine, The Children’s Hospital of Philadelphia, University of Pennsylvania, Philadelphia, PA USA; 3 Department of Pediatrics, University of Utah, Salt Lake City, UT USA; 4 Department of Pediatrics, Children’s National Hospital, George Washington University School of Medicine, Washington, DC USA; 5 Department of Pediatrics, Nemours/Alfred I. duPont Hospital for Children and Thomas Jefferson University, Wilmington, DE USA; 6 Department of Pediatrics, Benioff Children’s Hospital, University of California, San Francisco, San Francisco, CA USA; 7 Department of Pediatrics, Children’s Hospital of Michigan, Central Michigan University, Detroit, MI USA; 8 Department of Pediatrics, University of Colorado School of Medicine and Children’s Hospital Colorado, Aurora, CO USA; 9 Department of Critical Care Medicine, UPMC Children’s Hospital of Pittsburgh, University of Pittsburgh, Pittsburgh, PA USA; 10 Department of Pediatrics and Human Development, Michigan State University, Grand Rapids, MI USA; 11 Department of Pediatrics, Mattel Children’s Hospital, University of California Los Angeles, Los Angeles, CA USA; 12 Department of Pediatrics, Washington University School of Medicine, St. Louis, MO USA; 13 Department of Pediatrics, University of Arkansas for Medical Sciences and Arkansas Children’s Hospital, Little Rock, AR USA; 14 Department of Molecular Biology, Princeton University, Princeton, NJ USA

## Abstract

**Background:**

Resuscitation with chest compressions and positive pressure ventilation in Bidirectional Glenn (BDG) or Fontan physiology may compromise passive venous return and accentuate neurologic injury. We hypothesized that arterial pressure and survival would be better in BDG than Fontan patients.

**Methods:**

Secondary analyses of the Pediatric Intensive Care Quality of CPR and Improving Outcomes from Pediatric Cardiac Arrest databases. *P*-values were considered significant if < 0.05.

**Results:**

In total, 64 patients had either BDG (42/64, 66%) or Fontan (22/64, 34%) anatomy. Return of spontaneous circulation was achieved in 76% of BDG patients versus 59% of Fontan patients and survival with favorable neurologic outcome in 22/42 (52%) BDG versus 6/22 (27%) Fontan patients, *p* = 0.067. Twelve of 24 (50%) BDG and 2/7 (29%) Fontan patients who survived to discharge suffered new morbidity as defined by worsening Functional Status Score. More BDG patients achieved adequate DBP (≥25 mmHg for neonates and infants; ≥ 30 mmHg for children) than Fontan patients (21/23 (91%) vs. 5/11 (46%), *p* = 0.007).

**Conclusions:**

Only 27% of Fontan patients survived to hospital discharge with favorable neurologic outcome after CPR, likely driven by inadequate diastolic blood pressure during resuscitation. One half of the BDG patients who survived to hospital discharge had new neurologic morbidity.

**Impact statement:**

- Hemodynamic waveforms from 2 large prospective observational studies now allow for exploration of physiology during cardiopulmonary resuscitation for unique anatomy associated with single ventricle congenital heart disease.
- Fewer patients with Fontan physiology (46%) achieved an adequate diastolic blood pressure (defined as ≥ 25 mmHg for neonates and infants and ≥ 30 mmHg for children) than bidirectional Glenn patients during cardiopulmonary resuscitation (91%, *p* = 0.007).

- Only 27% of Fontan patients survived to hospital discharge with favorable neurologic outcome after cardiopulmonary resuscitation.
- Of the bidirectional Glenn patients who survived, 50% developed a new morbidity as quantified by the Functional Status Score.

## BACKGROUND

Cardiac arrest in patients with congenital heart disease (CHD) may present challenges to resuscitation based on the unique cardiovascular physiology resulting from surgical palliation. Recent resuscitation guidelines for CHD patients highlight the lack of data surrounding these special patient populations.^1^ Univentricular heart disease is palliated by a series of cardiac surgeries that stepwise result in passive pulmonary perfusion from the systemic venous system directly to the pulmonary vascular bed. The bidirectional Glenn (BDG) palliation directly anastomoses the superior vena cava (SVC) to the pulmonary arterial system and leaves normal inferior vena cava (IVC) venous return to the heart.^2^ The Fontan palliation baffles IVC flow directly to the pulmonary vascular bed which relieves cyanosis due to right to left shunting, but requires systemic ventricular preload to be directly dependent upon pulmonary vascular resistance and intrathoracic pressures.^3^ The unique physiology in patients with BDG and Fontan palliations can place cerebral circulation at particular risk during cardiac arrest due to higher baseline SVC pressures. In each circulation, venous return from the SVC must cross the pulmonary vascular bed passively prior to reaching the heart. In addition to the low flow state of perfusion during cardiac arrest, common resuscitative measures (e.g., chest compressions, positive pressure ventilation) result in an elevation of intrathoracic pressure that impedes this venous return, further compromising cerebral perfusion pressure. In patients with BDG and Fontan circulation, cardiac preload is also partially or fully dependent upon passive transpulmonary circulation.^4–6^ Similarly, resuscitative measures can compromise preload and cardiac output more than in normal two ventricle cardiac physiology.

There are minimal published data related to the resuscitation outcomes of the BDG and Fontan patient populations, and no multicenter reports of hemodynamics during cardiopulmonary resuscitation (CPR). The Collaborative Pediatric Critical Care Research Network has recently completed two prospective multicenter research studies in which comprehensive hemodynamic and clinical data surrounding resuscitation were obtained: Pediatric Intensive Care Quality of CPR (PICqCPR) and Improving Outcomes from Pediatric Cardiac Arrest **(**ICU-RESUS).^4,5^ Our aim was to describe outcomes in BDG and Fontan patient populations following CPR given the unique resuscitation hemodynamic datasets for complex physiology provided by these studies. Given partially preserved cardiac preload from IVC flow in the BDG circulation, we hypothesized that survival in BDG patients would be greater than Fontan patients. We also hypothesized that resuscitation hemodynamics and neurologic outcomes in BDG and Fontan patients following CPR would be worse than previously published populations.

## Methods

Both PICqCPR (July 2013–June 2016) and ICU-RESUS (October 2016–March 2021) were approved by the central IRB at the University of Utah with waiver of informed consent.^4,6^ Patients from both studies with BDG or Fontan physiology at the time of arrest were included. Subsequent arrest events and patients supported by extracorporeal membrane oxygenation (ECMO) at the time of arrest were excluded. A CONSORT Diagram is available in the supplemental materials (Supplemental Fig. 1). PICqCPR included patients who (1) received chest compressions for ≥ 1 min in the PICU or the CICU and (2) Age was ≥ 37 weeks gestational age and < 19 years of age. Patients were excluded in PICqCPR if they had (1) no arterial line or end tidal CO_2_ monitor in place at the time of chest compressions, (2) waveform data available from either the arterial line or ET CO2 monitor were not usable, or (3) at the time the CPR Event began, the patient was receiving ECMO therapy. ICU-RESUS enrolled subjects Age > 37 weeks and < 18 years of age and who received CPR in the ICU setting regardless of arterial line or end tidal CO_2_ monitor. ICU-RESUS excluded patients who had (1) a pre-existing terminal illness and patient was not expected to survive to hospital discharge, (2) there was a lack of commitment to aggressive ICU therapies, (3) had a brain death determination prior to CPR event, or (4) the first CPR event associated with this hospital admission was an out-of-hospital CPR event. Both studies had clinical data collection which utilized similar Utstein definitions; however, ICU-RESUS contained additional data fields specifically related to date of surgical intervention, presence of open chest, and Pediatric RISk of Mortality (PRISM) scores. Descriptive summaries of data fields for both study cohorts are included for reference (Supplemental Table 1). Hemodynamic and CPR quality data were processed using the same methodologies for both studies and was limited to the initial 10 min of arrest.^4,7,8^ Adequate values for age-specific blood pressure targets were examined based upon previous publications (diastolic blood pressure (DBP) ≥ 25 mmHg for neonates and infants and ≥ 30 mmHg for children, systolic blood pressure(SBP) ≥ 60 mmHg for neonates and infants and ≥ 80 mmHg for children).^4,9^

The primary outcome was survival with favorable neurologic outcome, defined as no more than moderate disability ( ≤ 3) or no increase from baseline Pediatric Cerebral Performance Category (PCPC) for patients with a baseline PCPC of 4 or 5. Secondary outcomes included the immediate outcome of CPR (i.e. return of spontaneous circulation (ROSC) for ≥ 20 min, transition to ECMO or death), development of new morbidity (defined as functional status score (FSS) change ≥3), differences in between those with and without survival with favorable neurologic outcome, and hemodynamic and CPR quality differences achieved in BDG or Fontan physiology. We additionally compared a more liberal endpoint of survival to hospital discharge irrespective of neurological status and a stricter definition of neurological favorable survival, defined as no more than mild disability (≤2) or no increase from baseline PCPC for patients with a baseline PCPC of 3, 4 or 5. Frequencies and percentages were reported for categorical variables. Medians, first quartiles, and third quartiles were reported for continuous variables. The univariable relationships with survival to hospital discharge were assessed using Fisher’s exact test for categorical variables and the Wilcoxon rank-sum test for ordinal variables. Analyses were performed using SAS 9.4 (SAS Institute; Cary, NC). There was no correction for multiple comparisons. All *p*-values were based on a two-sided alternative hypothesis and were considered significant if less than 0.05.

## Results

A total of 64 patients who underwent CPR with either BDG (42/64, 66%) or Fontan (22/64, 34%) anatomy had clinical data available for analysis. The predominant cardiac anatomic diagnosis for both BDG and Fontan patients was Hypoplastic Left Heart Syndrome (Supplemental Table 2). Table 1 compares pre-event characteristics between those who survived to hospital discharge and those who did not. Table 2 compares the event characteristics between survivors and non-survivors. Bradycardia with poor perfusion (38/64, 59%) was the most common initially documented rhythm. Non-survivors had more epinephrine doses administered than survivors (median 3 [2–7] vs. 2 [1,2] doses, *p* = 0.02). Importantly, most patients who arrested were not immediately post operative.

**Table 1:** Cohort and pre-event characteristics by survival to hospital discharge

		Survival to Hospital Discharge		
Variable	Overall (*N* = 64)	Yes (*N* = 31)	No (*N* = 33)	*P*-value
**Demographics**				
Age (years)	1.4 [0.5,3.6]	1.0 [0.4,3.1]	2.3 [0.6,8.1]	0.089^d^
Age category				0.051^c^
< 4 months	4 (6%)	2 (7%)	2 (6%)	
4 months - < 1 year	25 (39%)	14 (45%)	11 (33%)	
1 year - < 4 years	22 (34%)	13 (42%)	9 (27%)	
≥ 4 years	13 (20%)	2 (7%)	11 (33%)	
Male	40 (63%)	22 (71.0%)	18 (55%)	0.205^c^
Race				0.392^c^
White	31 (48%)	15 (48%)	16 (49%)	
Black or African American	21 (33%)	7 (23%)	14 (42%)	
Other	0 (0%)	0 (0%)	0 (0%)	
Unknown or Not Reported	12 (19%)	9 (29%)	3 (9%)	
**Preexisting medical conditions**				
Respiratory insufficiency	53 (83%)	24 (77%)	29 (88%)	0.331^c^
Hypotension	49 (77%)	22 (71%)	27 (82%)	0.382^c^
Congestive heart failure	17 (27%)	5 (16%)	12 (36%)	0.091^c^
Pneumonia	1 (2%)	0 (0%)	1 (3%)	1.000^c^
Sepsis	6 (9%)	4 (13%)	2 (6%)	0.419^c^
Trauma	1 (2%)	0 (0%)	1 (3%)	1.000^c^
Renal insufficiency	6 (9%)	2 (7%)	4 (12%)	0.673^c^
Pulmonary hypertension	18 (28%)	9 (29%)	9 (27%)	1.000^c^
**Characteristics before CPR event**				
Illness category				0.323^c^
Medical cardiac	18 (28%)	6 (19%)	12 (36%)	
Medical non-cardiac	2 (3%)	1 (3%)	1 (3%)	
Surgical cardiac	43 (67%)	23 (74%)	20 (61%)	
Surgical non-cardiac or trauma	1 (2%)	1 (3%)	0 (0%)	
Interventions in place				
Vascular access	63 (98%)	30 (97%)	33 (100%)	0.484^c^
Arterial catheter	48 (75%)	24 (77%)	24 (73%)	0.776^c^
Central venous catheter	51 (80%)	24 (77%)	27 (82%)	0.761^c^
Vasoactive infusion	53 (83%)	23 (74%)	30 (91%)	0.103^c^
Invasive mechanical ventilation	44 (69%)	20 (65%)	24 (73%)	0.592^c^
Non-invasive ventilation	10 (16%)	5 (16%)	5 (15%)	1.000^c^
End-tidal CO_2_ monitoring	36 (56%)	17 (55%)	19 (58%)	1.000^c^
PRISM^a^	6.0 [1.0,11.0]	7.0 [4.0,13.0]	3.0 [0.0,11.0]	0.136^d^
Baseline PCPC score^b^				0.567^d^
1 - Normal	26 (41%)	10 (32%)	16 (49%)	
2 - Mild disability	29 (45%)	18 (58%)	11 (33%)	
3 - Moderate disability	5 (8%)	3 (10%)	2 (6%)	
4 - Severe disability	4 (6%)	0 (0%)	4 (12%)	
5 - Coma/vegetative state	0 (0%)	0 (0%)	0 (0%)	
6 - Brain death	0 (0%)	0 (0%)	0 (0%)	
Baseline FSS^b^	8.0 [6.0,9.0]	8.0 [6.0,9.0]	8.0 [6.0,9.0]	0.962^d^

*PRISM* Pediatric RIsk of Mortality, *PCPC* Pediatric Cerebral Performace Category, *FSS* Functional Status Scale.

^a^PRISM was evaluated 2–6 h prior to the event.

^b^Baseline PCPC and FSS represent subject status prior to the event leading to the hospitalization.

^c^Fisher’s exact test.

^d^Wilcoxon rank-sum test.

**Table 2:** Event characteristics by survival to hospital discharge (Enrolled Index Events)

		Survival to Hospital Discharge		
Variable	Overall (*N* = 64)	Yes (*N* = 31)	No (*N* = 33)	*P*-value
**Characteristics of cardiac arrest event**				
Location of CPR Event				0.734^b^
PICU	10 (16%)	4 (13%)	6 (18%)	
CICU	54 (84%)	27 (87%)	27 (82%)	
CPR time^a^				1.000^b^
Weekday	35 (55%)	17 (55%)	18 (55%)	
Weeknight	12 (19%)	6 (19%)	6 (18%)	
Weekend	17 (27%)	8 (26%)	9 (27%)	
First documented rhythm				0.563^b^
Pulseless electrical activity / asystole	21 (33%)	9 (29%)	12 (36%)	
Ventricular fibrillation / tachycardia	4 (6%)	3 (10%)	1 (3%)	
Bradycardia with poor perfusion	38 (59%)	19 (61%)	19 (58%)	
Unknown	1 (2%)	0 (0%)	1 (3%)	
Duration of CPR (minutes)	6.5 [2.0,30.0]	6.0 [2.0,14.0]	8.0 [2.0,33.0]	0.402^c^
Duration of CPR (minutes)				0.071^b^
<6	29 (45%)	15 (48%)	14 (42%)	
6–15	12 (19%)	9 (29%)	3 (9%)	
16–35	10 (16%)	2 (7%)	8 (24%)	
>35	13 (20%)	5 (16%)	8 (24%)	
Sternum open at start of CPR event	9 (14%)	3 (10%)	6 (18%)	0.476^b^
Sternum opened during CPR event	3 (5%)	1 (3%)	2 (6%)	1.000^b^
Minutes to sternum opening	22.5 [18.0,27.0]	18.0 [18.0,18.0]	27.0 [27.0,27.0]	
Days since last cardiac surgery	18.0 [2.0,387.0]	18.0 [2.0,387.0]	20.5 [9.5,983.5]	0.377^c^
Immediate cause(s) of event				
Arrhythmia	9 (14%)	5 (16%)	4 (12%)	0.729^b^
Cyanosis without respiratory decompensation	3 (5%)	2 (7%)	1 (3%)	0.607^b^
Hypotension as immediate cause of event	37 (58%)	16 (52%)	21 (64%)	0.448^b^
Respiratory decompensation	38 (59%)	19 (61%)	19 (58%)	0.803^b^
**Pharmacologic interventions during arrest**				
Epinephrine	58 (91%)	29 (94%)	29 (88%)	0.673^b^
Number of doses	2.0 [1.0,5.0]	2.0 [1.0,2.0]	3.0 [2.0,7.0]	0.021^c^
Atropine	6 (9%)	2 (7%)	4 (12%)	0.673^b^
Calcium	29 (45%)	12 (39%)	17 (52%)	0.327^b^
Sodium bicarbonate	32 (50%)	12 (39%)	20 (61%)	0.133^b^
Vasopressin	5 (8%)	1 (3%)	4 (12%)	0.356^b^
Amiodarone	6 (9%)	1 (3%)	5 (15%)	0.198^b^
Lidocaine	1 (2%)	0 (0%)	1 (3%)	1.000^b^
Fluid bolus	15 (23%)	6 (19%)	9 (27%)	0.560^b^

*PICU* pediatric intensive care unit, *CICU* cardiac intensive care unit, *CPR* cardiopulmonary resuscitation.

^a^Weekday is between 7 AM and 11 PM Monday–Friday; weeknight is after 11 PM Monday–Thursday; Weekend is from 11 PM on Friday through 7 AM on the following Monday.

^b^Fisher’s exact test.

^c^Wilcoxon rank-sum test.

Outcomes of the entire 64 patient cohort including both BDG and Fontan patient groups are provided in Table 3. For our primary outcome, survival with favorable neurologic outcome (defined as PCPC score of 1, 2, 3, or no worse than baseline), we did not identify a statistical difference between the BDG and Fontan patient groups (*p* = 0.067) despite the higher absolute percentages of BDG patients than Fontan patients that survived (52% (22/42) vs. 27% (6/22), respectively). ROSC was achieved in 76% of BDG patients and 59% of Fontan patients, with hospital survival of 57% of BDG patients and 32% of Fontan patients (*p* = 0.069). Twelve of 24 (50%) Glenn and 2/7 (29%) Fontan patients (*p* = 0.412) who survived to discharge suffered new morbidity as defined by an increase in Functional Status Scale (FSS) score of at least 3 points. Importantly, when applying our alternative definition of neurologically favorable survival of a PCPC of 1,2 or no worse than baseline, we demonstrated better survival in BDG than Fontan patients (19/42 (42%) vs. 3/22 (14%), *p* = 0.013). Additionally, patients with BDG had lower overall PCPC scores at hospital discharge than Fontan patients (*p* = 0.014).

**Table 3:** Summary of outcomes

		Surgical palliation		
Outcomes	Overall (*N* = 64)	Bi-directional Glenn (*N* = 42)	Fontan (*N* = 22)	*P*-value
**Immediate outcome of CPR event**				0.098^c^
ROSC ≥ 20 min	45 (70%)	32 (76%)	13 (59%)	
Transitioned to ECMO	12 (19%)	8 (19%)	4 (18%)	
Died	7 (11%)	2 (5%)	5 (23%)	
**Hospital Discharge Outcomes**				
Survival to hospital discharge^a^	31 (48%)	24 (57%)	7 (32%)	0.069^c^
Survival to hospital discharge with favorable neurologic outcome	28 (44%)	22 (52%)	6 (27%)	0.067^c^
Survival to hospital discharge with PCPC of 1, 2, or no worse than baseline	22 (34%)	19 (45%)	3 (14%)	0.013^c^
PCPC at hospital discharge				0.014^d^
1 - Normal	6 (9%)	6 (14%)	0 (0%)	
2 - Mild disability	14 (22%)	12 (29%)	2 (9%)	
3 - Moderate disability	8 (13%)	4 (10%)	4 (18%)	
4 - Severe disability	3 (5%)	2 (5%)	1 (5%)	
5 - Coma/vegetative state	0 (0%)	0 (0%)	0 (0%)	
6 - Death	33 (52%)	18 (43%)	15 (68%)	
New morbidity (survivors only)^b^	14/31 (45%)	12/24 (50%)	2/7 (29%)	0.412^c^

*ROSC* Return of Spontaneous Circulation, *ECMO* ExtraCorporeal Membrane Oxygenation, *PCPC* Pediatric Cerebral Performance Category.

^a^Favorable neurologic outcome is defined as no more than moderate disability or no worsening from baseline Pediatric Cerebral Performance Category (PCPC).

^b^New morbidity among survivors is defined as a worsening from baseline FSS by 3 points or more.

^c^Fisher’s exact test.

^d^Wilcoxon rank-sum test.

Hemodynamic waveform data was available for 34/64 (53%) patients and is summarized in Table 4. This data included 23/42 (55%) BDG patients and 11/22 (50%) Fontan patients. We did not identify a difference in the diastolic blood pressure (DBP), systolic blood pressure (SBP), or achievement of previously established age-specific pressure targets (DBP ≥ 25 mmHg for neonates and infants and ≥ 30 mmHg for children, SBP ≥ 60 mmHg for neonates and infants and ≥ 80 mmHg for children) when comparing survivors to non-survivors. Additionally, we did not identify a difference in CPR quality metrics for ventilation rate, chest compression rate, or chest compression fraction. We also did not identify differences between survivors and non-survivors when examining the hemodynamic and quality measurements of resuscitation when limiting the comparisons to those patients with either BDG anatomy or Fontan anatomy (Supplemental Table 3).

**Table 4:** Hemodynamics and respiratory parameters by survival to hospital discharge

		Survival to hospital discharge		
Variable	Overall (*N* = 34)	Yes (*N* = 20)	No (*N* = 14)	*P*-value
**Average over (up to) the first 10** **min**				
DBP (mmHg)	34.1 [30.0,47.4]	38.1 [32.2,47.3]	31.9 [27.3,47.4]	0.327^b^
Adequate DBP^c^	26 (77%)	17 (85%)	9 (64%)	0.228^a^
SBP (mmHg)	79.5 [53.1,110.8]	83.3 [53.4,109.1]	72.8 [53.1,110.8]	0.713^b^
Adequate SBP^d^	20 (59%)	13 (65%)	7 (50%)	0.487^a^
ETCO2	28.3 [17.4,30.8]	28.3 [15.8,29.1]	31.7 [19.1,44.4]	0.617^b^
Ventilation rate (breaths per min)	24.1 [20.8,36.0]	27.3 [20.8,36.0]	22.0 [16.0,37.1]	0.699^b^
Chest compression rate (per min)	115.0 [104.1,124.0]	112.6 [100.3,118.7]	118.8 [106.7,134.5]	0.334^b^
Chest compression fraction	0.94 [0.89,0.98]	0.93 [0.90,0.97]	0.96 [0.88,0.99]	0.622^b^

The population for this table is Glenn/Fontan subjects with usable arterial waveform data; *DBP* diastolic blood pressure, *SBP* systolic blood pressure, *ETCO2* end-tidal carbon dioxide.

^a^Fisher’s exact test.

^b^Wilcoxon rank-sum test.

^c^DBP is considered adequate if ≥ 25 mmHg for neonates and infants and ≥ 30 mmHg for children.

^d^SBP is considered adequate if ≥ 60 mmHg for neonates and infants and ≥ 80 mmHg for children.

Table 5 compares resuscitation hemodynamics and quality measures between BDG and Fontan patients. BDG patients achieved a mean DBP of 39.7 [31.8, 47.4] mmHg and Fontan patients achieved a mean DBP of 28.8 [19.1, 47.4] mmHg, *p* = 0.063. A greater percentage of BDG patients achieved adequate DBP ( ≥ 25 mmHg for neonates and infants and ≥ 30 mmHg for children > 1 year of age) compared to Fontan patients (21/23 (91%) vs. 5/11 (46%), *p* = 0.007). We did not identify a difference between BDG and Fontan patients in the number of doses of epinephrine received (2.0 [1.0,5.0] vs. 2.0 [1.0,4.0], *p* = 0.52 respectively).

**Table 5:** Hemodynamics and respiratory parameters by Glenn vs. Fontan

		Surgical palliation		
Variable	Overall (*N* = 34)	Bi-directional Glenn (*N* = 23)	Fontan (*N* = 11)	*P*-value
**Average over (up to) the first 10** **min**				
DBP (mmHg)	34.1 [30.0,47.4]	39.7 [31.8,47.4]	28.8 [19.1,47.4]	0.063^b^
Adequate DBP^c^	26 (77%)	21 (91%)	5 (46%)	0.007^a^
SBP (mmHg)	79.5 [53.1,110.8]	79.0 [54.3,114.3]	83.6 [48.9,104.0]	0.883^b^
Adequate SBP^d^	20 (59%)	14 (61%)	6 (55%)	1.000^a^
ETCO2	28.3 [17.4,30.8]	28.4 [19.1,32.4]	22.4 [15.8,29.0]	0.617^b^
Ventilation rate (breaths per min)	24.1 [20.8,36.0]	22.0 [17.2,37.1]	30.0 [24.1,36.0]	0.661^b^
Chest compression rate (per min)	115.0 [104.1,124.0]	116.9 [109.8,124.0]	106.7 [99.4,124.2]	0.294^b^
Chest compression fraction	0.94 [0.89,0.98]	0.96 [0.91,0.99]	0.89 [0.84,0.94]	0.019^b^

The population for this table is Glenn/Fontan subjects with usable arterial waveform data, *DBP* diastolic blood pressure, *SBP* systolic blood pressure, *ETCO2* end-tidal carbon dioxide.

^a^Fisher’s exact test.

^b^Wilcoxon rank-sum test.

^c^DBP is considered adequate if ≥ 25 mmHg for neonates and infants and ≥ 30 mmHg for children.

^d^SBP is considered adequate if ≥ 60 mmHg for neonates and infants and ≥ 80 mmHg for children.

## Discussion

Our study quantified the survival to hospital discharge after cardiac arrest in patients with BDG and Fontan physiology and has demonstrated that: (1) BDG patients are more likely to attain adequate DBP thresholds during chest compressions than Fontan patients, (2) there was a trend for patients with BDG to have higher rates of survival with favorable neurological outcomes, defined as a PCPC of ≤ 3 or no worse than baseline than patients with Fontan physiology (BDG 52% (22/42) vs. Fontan 27% (6/22), *p* = 0.067), and (3) many survivors had new morbidities based on FSS (BDG 12/24 (50%) vs. Fontan 2/7 (29%), *p* = 0.412). These two large data sets, when combined, provide the largest available cohort for exploring CPR mechanics, hemodynamics, and outcomes in the unique physiology in BDG and Fontan patients. These data highlight that cardiopulmonary resuscitation of these patients presents unique challenges, in particular the need for all (Fontan) or part (Glenn) of the systemic venous return to passively cross the pulmonary vascular bed prior to reaching the ventricle as its preload. Such passive perfusion is impaired by the increased intrathoracic pressures generated by chest compressions and positive pressure ventilation applied during CPR [2,3] In addition, while during spontaneous circulation these physiologies are characterized by absence of a chamber pumping blood across the pulmonary bed, during CPR they are characterized by absence of a storage chamber which can fill with blood and then be compressed, meaning that venous return will be poorly converted to cardiac output.

Most outcome studies for cardiac arrest in patients with congenital heart disease have only examined outcomes in the postoperative period, and often did not differentiate specific physiologies. Reports from both the Pediatric Cardiac Critical Care Consortium (PC^4^) and the American Heart Association’s Get with the Guidelines-Resuscitation registry demonstrate hospital survival following cardiac arrest for pediatric cardiac patients were similar to other pediatric populations at 53 and 51%.^10,11^ However, neither report provided an assessment of neurologic status or new morbidity in survivors. Compared to other cardiac physiologies, patients with BDG and Fontan physiology rarely arrest in the perioperative period, which has hampered the ability to isolate the impact of this physiology on outcomes from CPR. A recent review of the Society of Thoracic Surgeons data demonstrated that only 17/1923 (0.9%) post-operative Fontan patients arrested with a survival of 7/17 (41%) in the immediate surgical period.^12^ We have demonstrated a rate of survival with favorable neurologic outcome in BDG patients (57%) comparable to the larger population of pediatric patients with congenital heart disease, but a survival rate of only 32% for patients with Fontan physiology, likely because we did not limit our observation to the immediate perioperative period which may have more readily reversible etiologies, such as cardiac tamponade.

Our study begins to examine the factors that may result in poor outcomes in Fontan patients following cardiac arrest. Achieving DBP targets has previously been associated with improved survival with favorable neurologic outcome in pediatric patients suffering cardiac arrest.^4^ Additionally, in patients with CHD, achieving DBP ≥ 25 mmHg for neonates and infants and ≥ 30 mmHg for children is associated with survival in surgical cardiac patients, but not for medical cardiac patients.^13^ We have demonstrated that Fontan patients are less likely to achieve these thresholds than BDG patients. Additionally, there was a very strong trend toward a lower DBP in Fontan anatomy, despite an older cohort of Fontan patients due to the sequential surgical palliation which occurs for single ventricle patients. We postulate that this superior hemodynamic profile may be due to partially preserved cardiac preload for BDG patients given that the IVC remains connected to the right atrium. Conversely, the entire cardiac preload in a Fontan patient must passively traverse the pulmonary vascular bed, and thus chest compressions and positive pressure ventilation may create significant hemodynamic impairments that are more difficult to overcome.

Despite achievement of ROSC and survival, cerebral perfusion may be impaired by the acute elevations in intrathoracic pressure occurring during CPR in these unique physiologies. As the SVC is connected surgically to the pulmonary circulation in both BDG and Fontan physiologies, cerebral venous pressures are elevated at baseline, placing cerebral circulation at greater risk for injury than in biventricular cardiac physiology due to inadequate cerebral perfusion during resuscitation. We have characterized cerebral injury in Fontan and BDG arrest survivors using both the PCPC and FSS. The majority of survivors met our definition of favorable neurological outcome (PCPC of 1, 2, 3, or no worse than baseline). However, some investigators have argued that a favorable neurological outcome is better defined by a PCPC score of 1,2, or no worse than baseline. With this stricter definition, the percentage of survivors with favorable neurologic outcome is lower (with only 45% of BDG patients and 14% of Fontan patients meeting this more stringent criteria), but there is also an association with better survival for BDG than Fontan patients (*p* = 0.014). Additionally, the resulting new morbidity in survivors, as defined by FSS score, of 29% for Fontan patients was similar to the overall patient cohorts for PICqCPR (29% (22/77)) and ICU-RESUS (31% (76/242)) survivors.^7,9^ In contrast, 50% of the BDG patients who survived had new morbidity. This would suggest that the preserved ventricular preload in the Glenn physiology may be beneficial for survival to hospital discharge, but the baseline elevated SVC pressures coupled with the impact on intrathoracic pressure from chest compressions and positive pressure ventilation may result in more frequent neurologic injury.

Both BDG and Fontan physiologies may benefit from methods of chest compression or ventilation that lower intrathoracic pressure to promote venous return. Computed tomography scan analysis of single ventricle patients demonstrated the largest cross sectional area of the systemic ventricle was under the lower quarter of the sternum, emphasizing the importance of hand positioning during standard CPR.^14^ Interposed abdominal compressions, a means of actively returning preload from the abdominal capacitance vessels to the thoracic cavity, has also been reported in Fontan physiology in a case report.^15^ Other adjunct compression techniques, such as active compression-decompression CPR using a suction cup on the chest that creates negative intrathoracic pressure, may theoretically improve hemodynamics in this physiology but remain untested for BDG and Fontan patients.^16,17^ Although not collected in this data set, ventilation strategy can play an important role in augmenting cardiac output. Ventilation with a larger tidal volume and lower rate can allow more time for passive pulmonary blood flow and avoidance of hyperventilation may result in increased cerebral blood flow.^1,18^ Additionally, respiratory devices that promote negative intrathoracic pressures could theoretically aid pulmonary blood flow in this physiology.^19–21^ Large animal models of Fontan physiology have only recently been developed, which may allow for investigation into these novel techniques.^22^ Our work helps to guide additional resuscitation studies by highlighting the hemodynamic challenges during CPR in human subjects with cavopulmonary connection physiology as well as the survival gap for Fontan patients and risks for development of new morbidity for BDG patients.

The most notable limitation of our study is that we performed a secondary analysis of two previous studies with variation in inclusion/exclusion criteria. Nevertheless, the studies were performed in many of the same centers with similar data collection, data definitions, and consistent analyses of hemodynamic and waveform data elements from the same core lab. We had additional limitations in that not all arterial waveform data were available or suitable for analysis resulting in a smaller population to explore the impact of hemodynamics on outcomes. Our database did not contain echocardiographic or cardiac catheterization data and thus the degree of prearrest ventricular dysfunction, atrioventricular valve regurgitation, or the presence of a fenestration in Fontan patients are unknown. The impact of these factors on survival is unknown, but we surmise they would have had minimal impact on the hemodynamics during resuscitation. Additionally, central venous line pressure tracings were not available for analysis, nor were results of brain imaging or other quantifications of neurologic injury, which may provide additional insight into this unique physiology.

## Conclusion

Providing physiologically adequate CPR for pediatric single ventricle patients palliated with BDG or Fontan physiology is challenging. Only 27% of Fontan patients survived to hospital discharge with favorable neurologic outcome, likely driven by difficulty in achieving diastolic blood pressure targets during resuscitation. Although 57% of BDG patients survived, 1/2 of these survivors had new morbidity as measured by FSS.

## Supplementary information

Supplementary figure 1

Supplementary table 3

Supplementary table2

Supplementary table 1

## Data Availability

The dataset from the ICU Resuscitation trial is available for public use via the National Institutes of Health (NIH) Biologic Specimen and Data Repository Information Coordinating Center (BioLINCC). The dataset from PICU-CPR is available for public use at https://www.cpccrn.org/study-datasets/.
