# Publisher Correction: Resuscitation arterial waveform quantification and outcomes in pediatric bidirectional Glenn and Fontan patients

**DOI:** 10.1038/s41390-024-03749-5

**Published:** 2024-11-26

**Authors:** Andrew R. Yates, David A. Hehir, Ron W. Reeder, John T. Berger, Richard Fernandez, Aisha H. Frazier, Kathryn Graham, Patrick S. McQuillen, Ryan W. Morgan, Vinay M. Nadkarni, Maryam Y. Naim, Chella A. Palmer, Heather A. Wolfe, Robert A. Berg, Robert M. Sutton, ICU-RESUS, Andrew R. Yates, David A. Hehir, Ron W. Reeder, John T. Berger, Richard Fernandez, Aisha H. Frazier, Kathryn Graham, Patrick S. McQuillen, Ryan W. Morgan, Vinay M. Nadkarni, Maryam Y. Naim, Chella A. Palmer, Heather A. Wolfe, Robert A. Berg, Robert M. Sutton, Tageldin Ahmed, Michael J. Bell, Robert Bishop, Matthew Bochkoris, Candice Burns, Joseph A. Carcillo, Todd C. Carpenter, J. Michael Dean, J. Wesley Diddle, Myke Federman, Ericka L. Fink, Deborah Franzon, Stuart H. Friess, Mark Hall, Christopher M. Horvat, Leanna L. Huard, Tensing Maa, Arushi Manga, Kathleen L. Meert, Peter M. Mourani, Daniel Notterman, Murray M. Pollack, Anil Sapru, Carleen Schneiter, Matthew P. Sharron, Neeraj Srivastava, Sarah Tabbutt, Bradley Tilford, Shirley Viteri, David Wessel, Athena F. Zuppa, the Eunice Kennedy Shriver National Institute of Child Health and Human Development Collaborative Pediatric Critical Care Research Network Investigator Groups, Andrew R. Yates, Ron W. Reeder, John T. Berger, Kathryn Graham, Patrick S. McQuillen, Robert A. Berg, Robert M. Sutton, Michael J. Bell, Joseph A. Carcillo, Todd C. Carpenter, J. Michael Dean, Ericka L. Fink, Mark Hall, Kathleen L. Meert, Peter M. Mourani, Daniel Notterman, Murray M. Pollack, Anil Sapru, David Wessel, Athena F. Zuppa

**Affiliations:** 1 Department of Pediatrics, Nationwide Children’s Hospital, The Ohio State University, Columbus, OH USA; 2 Department of Anesthesiology and Critical Care Medicine, The Children’s Hospital of Philadelphia, University of Pennsylvania, Philadelphia, PA USA; 3 Department of Pediatrics, University of Utah, Salt Lake City, UT USA; 4 Department of Pediatrics, Children’s National Hospital, George Washington University School of Medicine, Washington, DC USA; 5 Department of Pediatrics, Nemours/Alfred I. duPont Hospital for Children and Thomas Jefferson University, Wilmington, DE USA; 6 Department of Pediatrics, Benioff Children’s Hospital, University of California, San Francisco, San Francisco, CA USA; 7 Department of Pediatrics, Children’s Hospital of Michigan, Central Michigan University, Detroit, MI USA; 8 Department of Pediatrics, University of Colorado School of Medicine and Children’s Hospital Colorado, Aurora, CO USA; 9 Department of Critical Care Medicine, UPMC Children’s Hospital of Pittsburgh, University of Pittsburgh, Pittsburgh, PA USA; 10 Department of Pediatrics and Human Development, Michigan State University, Grand Rapids, MI USA; 11 Department of Pediatrics, Mattel Children’s Hospital, University of California Los Angeles, Los Angeles, CA USA; 12 Department of Pediatrics, Washington University School of Medicine, St. Louis, MO USA; 13 Department of Pediatrics, University of Arkansas for Medical Sciences and Arkansas Children’s Hospital, Little Rock, AR USA; 14 Department of Molecular Biology, Princeton University, Princeton, NJ USA

Correction to: *Pediatric Research* 10.1038/s41390-024-03564-y, published online 16 September 2024

Due to a typesetting mistake, the first paragraph of the main text was inadvertently included as part of the abstract. The original article has been corrected.

