# Supplementary figures and images for: Resuscitation arterial waveform quantification and outcomes in pediatric bidirectional Glenn and Fontan patients
Source: Pediatr Res. 2024 Sep 16;97(6):1989–96. doi: 10.1038/s41390-024-03564-y (PMC12122355; doi:10.1038/s41390-024-03564-y)

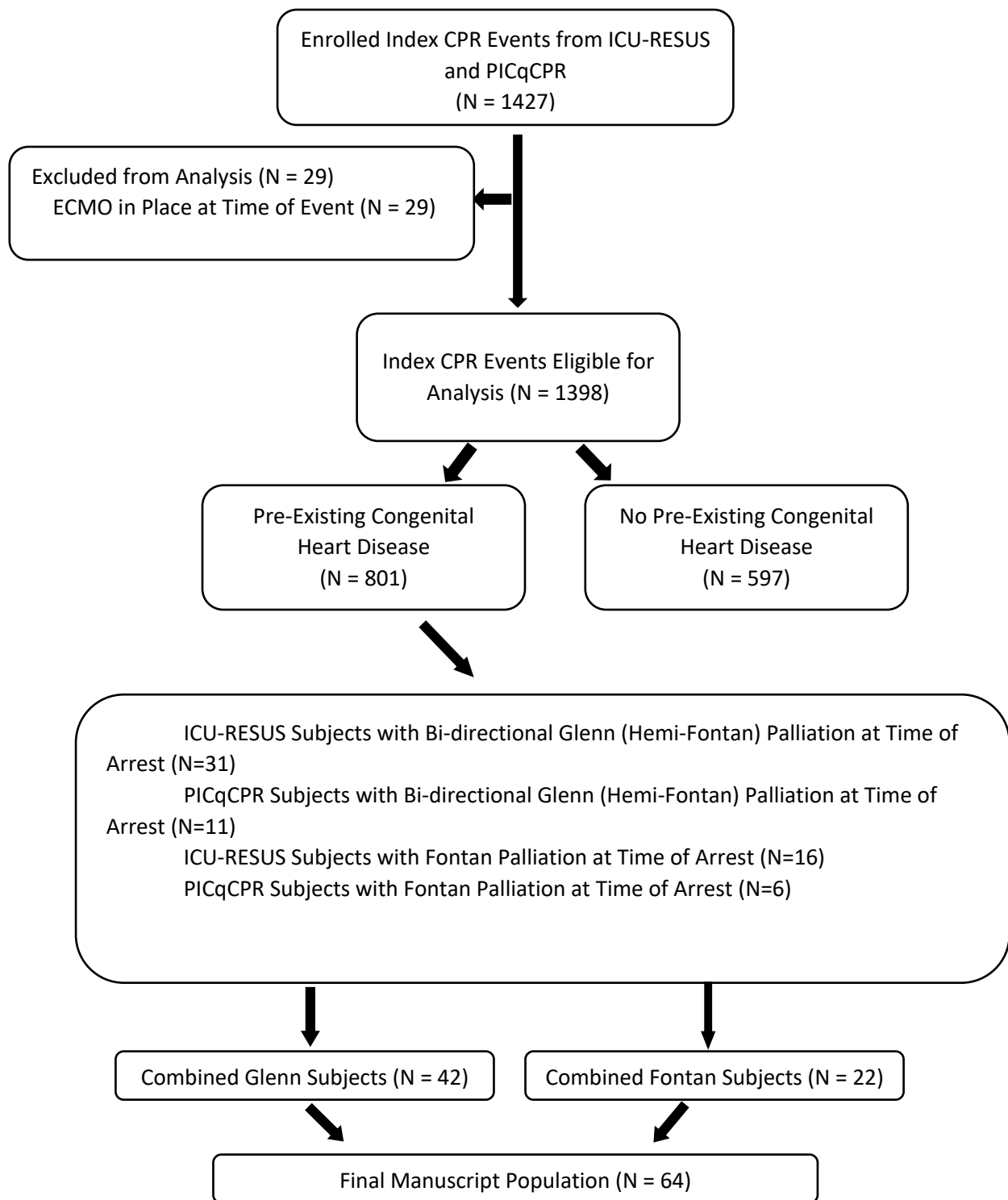

Supplement: Supplementary file 1 — Supplementary figure 1 [file 41390_2024_3564_MOESM1_ESM.pdf]
