# Supplementary material for: Resuscitation arterial waveform quantification and outcomes in pediatric bidirectional Glenn and Fontan patients
Source: Pediatr Res. 2024 Sep 16;97(6):1989–96. doi: 10.1038/s41390-024-03564-y (PMC12122355; doi:10.1038/s41390-024-03564-y)
Supplement: Supplementary file 2 — Supplementary table 3 [file 41390_2024_3564_MOESM2_ESM.pdf]

Supplemental Table 3.

Glenn or Fontan Hemodynamics and respiratory parameters by survival to hospital discharge

		Survival to hospital discharge		
	Overall	Yes	No	P-value
**Glenn subjects only**	N = 23	N = 14	N = 9	
**Average over (up to) the first 10 minutes**				
DBP (mmHg)	39.7 [31.8,47.4]	43.4 [35.2,47.4]	32.3 [30.0,40.2]	0.139[2]
Adequate DBP[3]	21 (91.3%)	14 (100.0%)	7 (77.8%)	0.142[1]
SBP (mmHg)	79.0 [54.3,114.3]	81.5 [54.3,114.3]	68.0 [54.8,110.8]	0.682[2]
Adequate SBP[4]	14 (60.9%)	9 (64.3%)	5 (55.6%)	1.000[1]
ETCO2	28.4 [19.1,32.4]	28.4 [20.1,30.8]	31.7 [19.1,44.4]	0.817[2]
Ventilation rate (breaths per min)	22.0 [17.2,37.1]	25.6 [19.0,35.9]	22.0 [16.0,37.1]	0.860[2]
Chest compression rate (per min)	116.9 [109.8,124.0]	117.2 [110.5,121.5]	116.4 [109.8,124.0]	0.894[2]
Chest compression fraction	0.96 [0.91,0.99]	0.94 [0.91,0.97]	0.99 [0.94,1.00]	0.202[2]
**Fontan subjects only**	N = 11	N = 6	N = 5	
**Average over (up to) the first 10 minutes**				
DBP (mmHg)	28.8 [19.1,47.4]	30.0 [19.1,33.0]	28.8 [27.3,47.4]	0.927[2]
Adequate DBP[3]	5 (45.5%)	3 (50.0%)	2 (40.0%)	1.000[1]
SBP (mmHg)	83.6 [48.9,104.0]	92.6 [48.7,104.0]	77.6 [53.0,97.9]	1.000[2]
Adequate SBP[4]	6 (54.5%)	4 (66.7%)	2 (40.0%)	0.567[1]
ETCO2	22.4 [15.8,29.0]	22.4 [15.8,29.0]	- [-,-]	
Ventilation rate (breaths per min)	30.0 [24.1,36.0]	30.0 [24.1,36.0]	- [-,-]	
Chest compression rate (per min)	106.7 [99.4,124.2]	102.2 [99.4,109.3]	124.2 [106.7,134.5]	0.171[2]
Chest compression fraction	0.89 [0.84,0.94]	0.90 [0.84,0.94]	0.89 [0.88,0.90]	0.927[2]

The population for this table is Glenn subjects with usable arterial waveform data; DBP = diastolic blood pressure; SBP = systolic blood pressure; ETCO2 = end-tidal carbon dioxide.

[1] Fisher's exact test.

[2] Wilcoxon rank-sum test.

[3] DBP is considered adequate if ≥ 25 mmHg for neonates and infants and ≥ 30 mmHg for children.

[4] SBP is considered adequate if ≥ 60 mmHg for neonates and infants and ≥ 80 mmHg for children.
