# Supplementary material for: Resuscitation arterial waveform quantification and outcomes in pediatric bidirectional Glenn and Fontan patients
Source: Pediatr Res. 2024 Sep 16;97(6):1989–96. doi: 10.1038/s41390-024-03564-y (PMC12122355; doi:10.1038/s41390-024-03564-y)
Supplement: Supplementary file 3 — Supplementary table2 [file 41390_2024_3564_MOESM3_ESM.pdf]

Supplemental Table 2: Cardiac Anatomy in Bi-directional Glenn and Fontan patients

Variable	Overall (n=64)	Bi-directional Glenn (n=42)	Fontan (n=22)	p-value
**Cardiac Anatomy**				0.09[1]
Hypoplastic Left Heart Syndrome (HLHS)	32	20	12	
Double Outlet Right Ventricle (DORV)	9	9	0	
Tricuspid atresia	4	3	1	
Pulmonary atresia	3	2	1	
Other Single Ventricle	16	8	8	

[1]Fischer's Exact Test
